# Pharyngeal Pumping Assay for Quantifying Feeding Behavior in *Caenorhabditis elegans*

**DOI:** 10.21769/BioProtoc.5073

**Published:** 2024-09-20

**Authors:** Muniesh Muthaiyan Shanmugam, Pankaj Kapahi

**Affiliations:** Buck Institute for Research on Aging, Novato, CA, USA

**Keywords:** *C. elegans*, Pharyngeal pumping, Feeding, Worms, Microscopy, Stereomicroscope, Eating behaviors

## Abstract

*C. elegans* is a well-established nematode model organism, with 83% of its genes conserved in humans with translation potential. C. elegans is translucent, with clearly defined cellular organization, and robustly identifiable under a microscope, being an excellent model for studying feeding behavior. Its neuromuscular pharyngeal pump undergoes a pumping motion that can be quantified to study feeding behavior at specific treatment conditions and in genetically modified worms. Understanding the evolutionarily conserved feeding behaviors and regulatory signals is vital, as unhealthy eating habits increase the risk of associated diseases. The current protocol was developed to identify and study evolutionary conserved signals regulating feeding behavior. The protocol described here is very robust in calculating the pumping rate (pumping per minute) as it directly counts the pharyngeal pumping for 30 s. This protocol uses basic laboratory instrumentation, such as a stereomicroscope with an attached camera and a computer with a video program that can be used to count manually. The advantages of studying C. elegans feeding include understanding the genetic basis of feeding regulation, dysregulation of feeding behavior in a disease model, the influence of toxic or environmental substances in feeding behavior, and modulation of feeding behavior by pharmacological agents.

Key features

• Quantifies pharyngeal pumping, which can be used to study up/downstream signaling in feeding regulation.

• Uses a phenotype (pharyngeal pumping) that is easy to score.

• Requires only a stereomicroscope with a camera to record the pharyngeal pumping, which can be counted manually.

## Background

Feeding is a crucial and complex behavior that ensures the intake of nutrition for survival and healthy living across animal species [1]. With the recent availability of calorie-dense foods and an increase in unhealthy eating habits [2], it has become necessary to understand feeding behavior and the mechanisms that thoroughly regulate it. Discovery of the mechanisms regulating feeding behavior in primitive model organisms offers a faster understanding with translation potential to humans. *Caenorhabditis elegans* is a tiny translucent nematode worm, with only 1 mm in length, and a robust feeding behavior that is easy to quantify. These worms have a short life cycle (duration for the larval worms to reach the reproductive age, or young adults) of 3.5 days at 20 °C and a lifespan of approximately 30 days, making them cost-effective to maintain in a laboratory [3]. With an 83% gene homology to humans and a high translation potential, *C. elegans* is an excellent model organism for genetic manipulations to study various biological processes and discover previously unexplained signaling pathways [4]. The worms feed on bacteria through their pharynx, and their feeding rate depends on the neuromuscular pumping action of their pharyngeal muscles. The worm's intake of bacterial food is facilitated by two pharyngeal movements, namely pumps and isthmus peristalses. Pumping occurs due to cycles of coordinated contraction and relaxation of pharyngeal muscles, which can be measured to determine the feeding rate. Isthmus peristalsis movement involves the opening of the posterior lumen, followed by closure in an anterior-to-posterior wave [5,6].

**Figure 1.**
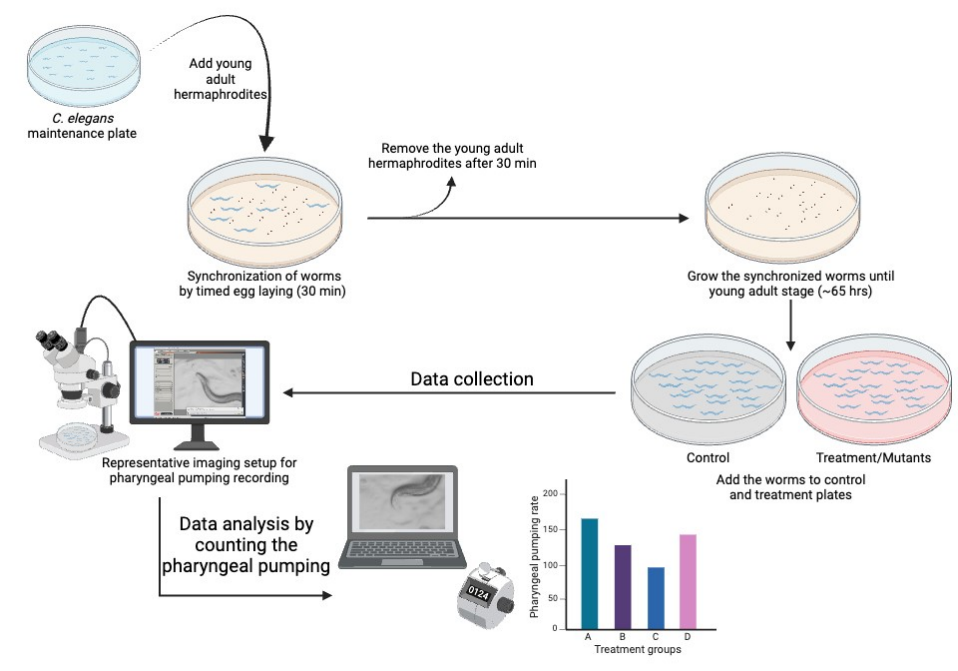
Step-by-step visual guide for quantifying pharyngeal pumping in *C. elegans* to study feeding behavior. Refer to the representative Video 1.

Extensive understanding of *C. elegans* feeding behaviors exists, and with availability of food ad libitum, the pharyngeal pumping rate is the limiting step for food intake [5–9]. The method outlined here is robust because it directly counts the pharyngeal pumping movement using a manual counter (Figure 1). To do this, a low-magnification stereomicroscope with an attached camera can be used to record the pumping of pharyngeal muscles in an unrestrained *C. elegans* in vivo for further analysis. Since the pumping rate is very high and difficult to follow with the naked eye, video player software can be used to slow down the speed, making it possible to count the pumps manually using a clicker counter [10]. However, implementing this method can be laborious for collecting and analyzing large datasets or conducting high-throughput analysis. Recently, Bonnard et al. [11] developed an automated system to measure unrestrained worms' pharyngeal pumping and foraging behavior, which can be implemented for conducting high-throughput analysis and screening. Further, a microfluidic device was developed to measure the pumping rate on restrained worms in microfluidic channels for high-throughput analysis [12]. Other methods for quantifying feeding behavior include measuring the reduction in bacterial density in liquid culture (bacterial clearance assay) [13,14], intestinal fluorescence of GFP-labeled bacteria, and BODIPY dye [6]. Compared to the alternative methods mentioned above, direct counting of pharyngeal pumping requires less standardization with basic equipment that commonly exists in any *C. elegans* laboratory and is quicker if a large number of samples is not involved.

## Materials and reagents

**Biological materials**

1. Required *C. elegans* strains (N2 wild-type and *glod-4* mutant). The *C. elegans* strains used in this study were obtained from the Caenorhabditis Genetic Center (CGC) in Minneapolis, USA, and a few other/necessary strains can be obtained from the National Bioresource Project, Tokyo, Japan. Please refer to wormbook.com for basic worm culture techniques
2. Bacteria *E. coli* OP50-1. Obtained from Caenorhabditis Genetic Center (CGC), Minneapolis, USA

**Reagents**

1. Sodium chloride (Sigma-Aldrich, catalog number: S9888)
2. Bacto^TM^ peptone (BD Diagnostic Systems, catalog number: 211677)
3. Agar (BD DIFCO^TM^, catalog number: 214510)
4. Calcium chloride (Sigma-Aldrich, catalog number: C3881)
5. Magnesium sulfate (Sigma-Aldrich, catalog number: 208094)
6. Cholesterol (Sigma-Aldrich, catalog number: C8667)
7. Streptomycin sulfate (Sigma-Aldrich, catalog number: S1567)
8. Nystatin (Chem Impex, catalog number: 00816)
9. Potassium phosphate monobasic (Sigma-Aldrich, catalog number: P0662)
10. Potassium phosphate dibasic (Sigma-Aldrich, catalog number: P3786)
11. Luria-Bertani (LB) broth (BD Difco^TM^, catalog number: 244610)
12. Serotonin hydrochloride (Sigma-Aldrich, catalog number: H9523)

**Solutions**

1. 1 M calcium chloride (autoclave to sterilize)
2. 1 M magnesium sulfate (autoclave to sterilize)
3. 5 mg/mL cholesterol in ethanol
4. 100 mg/mL streptomycin sulfate (filter with 0.2 μm filter to sterilize)
5. 10 mg/mL nystatin in ethanol
6. Potassium phosphate buffer: Mix 868 mL of potassium phosphate monobasic (1 M) and 132 mL of potassium phosphate dibasic (1 M), pH 6 (autoclave to sterilize)
7. 0.5 M serotonin hydrochloride stock
*Note: Double-distilled or MilliQ water is used as solvent unless specified.*
8. Nematode growth medium (NGM) plates (see Recipes)
9. LB broth (see Recipes)
10. OP50-1 culture (see Recipes)

**Recipes**

1. **Nematode growth medium (NGM) plates**
  Prepare 3 g of sodium chloride, 2.5 g of peptone, and 25 g of agar in 970 mL of water, then autoclave the solution (15 psi pressure at 121 °C for 30–60 min) to sterilize it. Once the solution has reached a bearable temperature (bearable when the container touches the ventral side of the arm below the hand), add 25 mL of potassium phosphate buffer and 1 mL each of 1 M magnesium sulfate, 1 M calcium chloride, cholesterol, streptomycin, and nystatin in a laminar flow hood. After thorough mixing, pour 25 mL of the solution into 10 cm Petri plates and allow it to cool inside the laminar flow hood overnight and solidify. Spread 500 µL of 5× concentrated OP50-1 bacterial culture on the NGM plates and incubate at 37 °C for 16 h. After 16 h, store the plates at 4 °C until used. Although the plates can be stored at 4 °C for a month if properly stored in a sealed box with reduced perspiration, use the plates within a week.
2. **LB broth**
  Dissolve 25 g of LB in 1 L of water in a 5 L conical flask and autoclave to sterilize the media. The autoclaved LB media can be stored at 4 °C for up to a month or until its sterility is compromised by outside contaminants.
3. **OP50-1 culture**
  Inoculate a colony of the OP50-1 *E. coli* strain in LB broth (1 L of LB broth prepared as in Recipe 2) containing streptomycin sulfate (working concentration of 100 μg/mL) using a sterile inoculating loop. OP50-1 obtained from CGC should be cultured, maintained, and stored at -80 °C. Streak the OP50-1 in an LB agar plate to isolate a single colony (refer to basic microbiological technique for maintenance of bacterial strain). Incubate the culture at 37 °C at 250 rpm in a bacterial incubator (shaking the culture at 250 rpm improves oxygenation, resulting in better bacterial growth) for 16 h. After 16 h, concentrate the bacterial culture five times by centrifugation at 4,000× *g* for 10 min in a 50 mL tube; remove the necessary supernatant (40 mL). Vortex (use maximum speed setting in the vortexer) the pellet to disperse the bacterial pellet, which can then be used to spread on the NGM plates (500 μL of bacterial culture/NGM plate).

**Laboratory supplies**

1. Glass conical flasks (5 L)
2. Measuring cylinder (1,000 mL and 500 mL)
3. Spatula
4. Disposable pipette (50 mL, 25 mL, and 10 mL) and motorized pipette controller (Accuhelp, model: PH01-B)
5. Pipette tips and micropipette (Eppendorf)
6. 10 cm disposable sterile plastic Petri plates (standard Petri dishes) (Celltreat^® ^Scientific Products, catalog number: 229695)
7. Inoculating loop (UltraCruz^®^, model: sc-200265)

## Equipment

1. Autoclave (any basic or benchtop autoclave that can reach 15 psi and 121 °C for 30 min)
2. Weighing balance (Ohaus, model: Pioneer PX163 analytical and precision balance)
3. Laminar flow hood
4. Stereomicroscope (Leica, model: M165 FC)
5. Computers for recording the pharyngeal pumping video and analysis (A basic computer with Windows operating system that runs Window Media Player or/and VLC media player)
6. Handheld manual counter clicker
7. Bacterial shaker incubator set to 37 °C and >250 rpm
8. Centrifuge (Eppendorf, model: 5810 R)
9. Overhead projector (OHP) sheets or multifunction transparency film (example: Optiazure, multifunction transparency film 8.5 × 11 inch)
10. WormStuff worm pick with platinum wire (Genesee Scientific, model: 59-AWP)
11. Worm incubator at 20 °C
12. Bacterial incubator at 37 °C (to grow OP50-1 on the NGM plates; Recipe 1)
13. Vortexer

## Software and datasets

1. Leica microscope software LAS V4.12
2. Window media player
3. VLC media player
4. Microsoft Excel
5. GraphPad Prism (any version)

## Procedure

- **Preparation of worms**
*Note: Worms are synchronized by timed egg-laying.*
  1. Transfer 35 young-adult hermaphrodite worms (per plate) to fresh NGM plates containing OP50-1 to lay embryos on the NGM plates. [Choose the number of plates based on the number of treatment groups for the experiment. Each group should have at least 30–40 worms (N = 30–40) for data collection.]
  2. Incubate the plates at 20 °C for 30 min.
  3. After 30 min, remove the adult worms from the plates. They can be transferred to another plate for further use or discarded.
  4. Incubate the plates with embryos at 20 °C until the worms reach the young-adult stage (approximately 65 h from timed egg-laying).
  5. For treatment, such as 5 mM serotonin, overlay the NGM plate containing OP50-1 bacteria (grown on 25 mL of solidified media) with the 250 μL of 500 mM serotonin stock solution. Rotate the NGM plate to ensure the stock solution spreads across the entire surface. Allow the spread stock solution to air dry in the laminar flow hood and diffuse into the agar to achieve the desired concentration.
  6. A necessary number of young-adult worms (N = 30–40) are transferred to either a treatment plate or control plate without the drug and incubated for an appropriate amount of time [e.g., for N2 vs. *glod-4* genetic mutant (Figure 2), capture videos of appropriately aged worms (no treatment necessary), 1 h incubation for serotonin (Figure 3) and 24 h for MG-H1 [10] (methylglyoxal-derived hydroimidazolone)].
*Note: Basic worm maintenance techniques are found in WormBook in the chapter “Maintenance of C. elegans” [15]*.
- **Data collection**
  1. We used a Leica M165 FC stereomicroscope to capture video recordings of pharyngeal pumping using a 2× objective lens with brightfield illumination. Any stereomicroscope with a 2× objective lens and a camera to record movies can be used to record pharyngeal pumping videos.
  2. To capture the worms' live activity, keep the exposure per frame very low, around 5 ms, and record movies for 40 s (Video 1).
  3. Choose a healthy, actively moving worm on the bacterial lawn, and start recording the videos. Do not record the worms that were harmed while picking (worms that cannot move in a sinusoidal waveform, worms that are not moving, and worms that are severely injured, like being split open near the vulva) or while transferring during treatment (if necessary, use eyelash picks to pick and transfer the worms). Worms outside the bacterial lawn should not be considered for data collection and analysis.
  4. Since the worms are unrestrained, they will move freely. Therefore, move the plate to keep the worm within the objective lens's field of view. To exert less stress on the hand while freely moving the plate to accommodate the worm in the field of view, place the Petri plates on the transparent plastic sheet (OHP sheets) and move the sheet accordingly to move the plate around (Figure 4).
  5. The obtained pharyngeal pumping recording can be analyzed as described below.

## Data analysis

1. Run the video obtained from the Leica M165 FC stereomicroscope on Windows Media Player Legacy software at 0.25 times the original speed (open the video → right-click → *Enhancements* → *Play speed settings* → reduce the speed to 0.25) and count manually using a handheld counter clicker until the video reaches 30 s of recording.
2. Enter counted values in either Microsoft Excel or GraphPad Prism for further statistical analysis. A Student *t*-test can be used to compare two experimental groups, and a one-way ANOVA can be used to compare more than two groups. In the examples provided with this protocol, we utilized a Student *t*-test to compare the experimental groups (Figures 2: N2 vs. *glod-4*, and Figure 3: untreated control vs. serotonin treatment). Please follow the instructions in the software manual to perform statistical analysis.

## Validation of protocol

1. This protocol has been used and validated in the following research article: Muthaiyan Shanmugam et al. [10]. Methylglyoxal-derived hydroimidazolone, MG-H1, increases food intake by altering tyramine signaling via the GATA transcription factor ELT-3 in *Caenorhabditis elegans—eLife* (Figure 2). The data below compares the pharyngeal pumping of N2 wild-type worms with that of *glod-4(gk189)* mutant worms. The glyoxalase enzyme (*glod-4*) is responsible for detoxifying methylglyoxal (MGO), which non-enzymatically interacts with biomolecules to form advanced glycation end-products (AGEs). The absence of the glyoxalase enzyme leads to increased accumulation of AGEs. MG-H1, a type of MGO-derived AGE, has been shown to increase feeding behavior in our research.
  **Figure 2.**
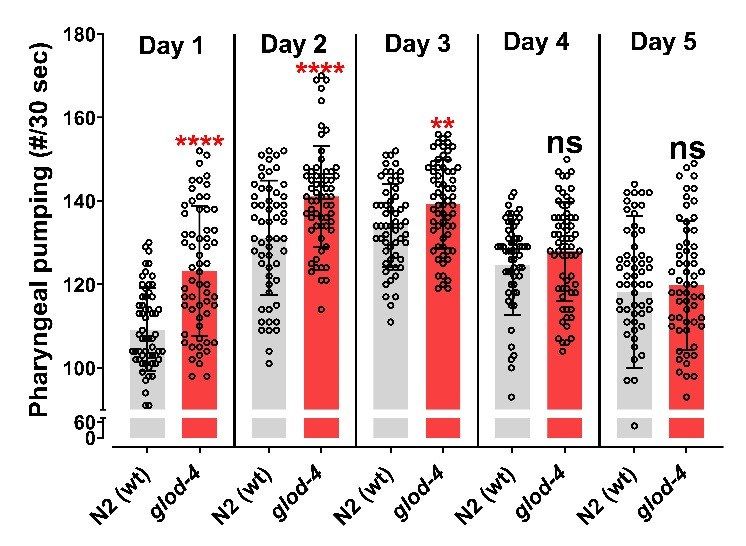
Glyoxalase *glod-4* mutant worms exhibit increased pharyngeal pumping. Quantification of pharyngeal pumping (number/30 s) in N2(wt) and *glod-4(gk189)* mutants at different stages of adulthood from day 1 of the young-adult stage (65 h after egg laying). Student *t*-test. ****p < 0.0001 and **p < 0.01. # represents number. Figure reprinted from Muthaiyan Shanmugam et al. [10]. The Creative Commons Attribution License permits unrestricted use of this article with proper acknowledgment to the authors and source.
2. Serotonin is a neurotransmitter that plays a vital role in regulating various behaviors in *C. elegans*, such as locomotion, pharyngeal pumping, or egg-laying [16]. We validated our method by quantifying pharyngeal pumping after treating day 1 young-adult N2 wild-type worms with 5 mM of serotonin for 1 h. The results demonstrate a significant increase in pharyngeal pumping following 1 hour of serotonin treatment (Figure 3).
  **Figure 3.**
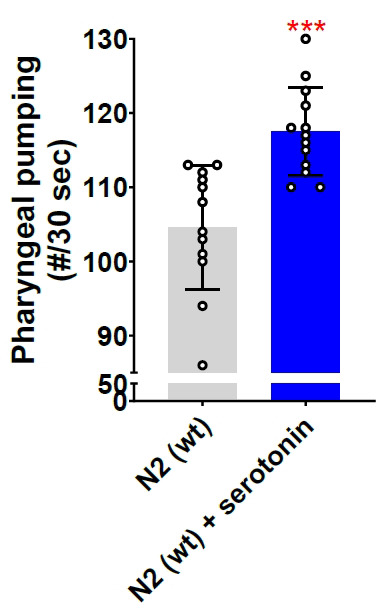
Serotonin neurotransmitter increases pharyngeal pumping. Quantification of pharyngeal pumping after 5 mM serotonin treatment for 1 h on N2 wild-type worm (day 1 young-adult worms, 65 h after egg laying). Equal solvent volume (in this case, water, as serotonin hydrochloride is dissolved in water) was added over the control NGM plates containing OP50-1. Student *t*-test. ***p < 0.001. # represents number.
3. The literature consistently shows that the pharynx pumps at a rate of 200–300 pumps per minute when food is available [5,6,14]. Our calculated pumping rate, as shown in Figures 2 and 3, aligns with the rates reported in the literature, further validating our approach.

## General notes and troubleshooting

1. The pharyngeal pumping rate varies with the worm's development [10] (Figure 2), so it is important to collect data from different treatment groups or genetic backgrounds at the exact developmental stage to compare them accurately. One way to achieve this is to synchronize the worm groups individually at a specific time rather than simultaneously, to ensure the exact developmental stage during data collection.
2. When designing data collection for large sample sizes or multiple test groups, it is important to consider the technician’s or researcher’s fatigue that may result from the technique used. It typically takes about a minute to locate an unrestrained worm, focus, and begin recording a 40 s pumping video, of which 30 s of video is available for manual counting to acquire data. Depending on the penetrance of the observed phenotype, it is necessary to collect data from at least 30 individual worms per group, which requires 40–50 min, depending on the fatigue experienced. Planning short resting periods during data collection between treatment groups is wise. Alternatively, when comparing multiple treatment or genetic groups, the video recording of worms from each group can be staggered. This means that 10 worms from each group are recorded, followed by worms from the other groups, and this cycle is repeated until the desired number of worms from each group has been recorded, thus minimizing any variations caused by the recording process.
3. To account for variations induced by lab temperature fluctuations, prepare three worm plates for each treatment group. Record data from only 10 worms per plate, while the other plates are incubated at 20 °C.
4. The video file generated by the Leica software LAS V4.12 is generally very large, which makes transferring raw data to another computer difficult and time-consuming. If necessary, the VLC media player can be used to record the desktop for 40 s when a worm is focused and projected using the Leica software LAS V4.12 in live mode (Video 1); while doing so, set 50 frames per second in the VLC media player option. This generates pumping videos with a smaller file size, which makes transferring files easier.
  **Video 1.**
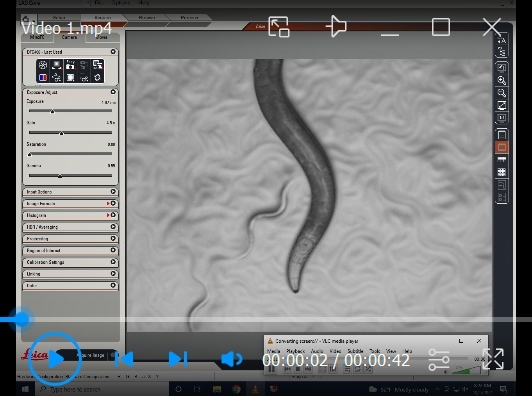
Representative video recording of an N2 wild-type worm as described in the protocol
5. To ensure that the unrestrained worms on the NGM plate containing OP50-1 remain in the field of the objective lens, it is necessary to move the NGM plate constantly. However, constantly holding and moving the NGM plate can exert considerable stress on the hand. One way to reduce this stress is to place the NGM plate on a transparent plastic sheet, like an overhead projector (OHP) sheet, and slide the sheet as the worms move to keep them in the field of the objective lens (Figure 4).
  **Figure 4.**
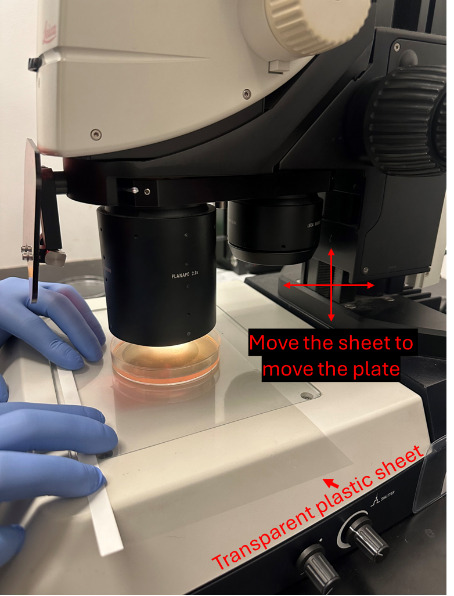
Picture showing the use of a transparent plastic sheet to move the NGM plate without exerting stress on the hand by holding the plate
